# Testbed as a RegUlatory Science Tool (TRUST): A Testbed Design for Evaluating 5G-Enabled Medical Devices

**DOI:** 10.1109/access.2023.3299242

**Published:** 2023

**Authors:** YONGKANG LIU, MOHAMAD OMAR AL KALAA

**Affiliations:** 1 Office of Science and Engineering Laboratories (OSEL), Center for Devices and Radiological Health (CDRH), U.S. Food and Drug Administration, Silver Spring, MD, USA; 2 Office of Science and Engineering Laboratories (OSEL), Center for Devices and Radiological Health (CDRH), U.S. Food and Drug Administration, Silver Spring, MD, USA

**Keywords:** Biomedical information, biomedical equipment, 5G mobile communication, testing

## Abstract

The fifth-generation (5G) cellular communication technology introduces technical advances that can expand medical device access to connectivity services. However, assessing the safety and effectiveness of emerging 5G-enabled medical devices is challenging as relevant evaluation methods have not yet been established. In this paper, we propose a design model for 5G testbed as a regulatory science tool (TRUST) for assessing 5G connectivity enablers of medical device functions. Specifically, we first identify application specific testing needs and general testing protocols. Next, we outline the selection and customization of key system components to create a 5G testbed. A TRUST demonstration is documented through a realistic 5G testbed implementation along with the deployment of a custom-built example use-case for 5G-enabled medical extended reality (MXR). Detailed configurations, example collected data, and implementation challenges are presented. The openness of the TRUST design model allows a TRUST testbed to be easily extended and customized to incorporate available resources and address the evaluation needs of different stakeholders.

## I. INTRODUCTION

The fifth-generation (5G) cellular communication technology has been rapidly deployed in major telecommunication markets worldwide. Compared to 4G, 5G promises to bring performance improvements for cellular communication (e.g., 10x increase in throughput) that can enhance user experiences of current mobile services and inspire innovative connectivity applications. 5G communications refer to the end-to-end service path, including radio frequency (RF) links and core network connections, between end users and remote application resources deployed either on the network edge or in the cloud. Early adoptions of 5G technology were driven by 5G equipment vendors and mobile network operators (MNO, also known as “operators” or “carriers”) and focused on the consumer electronics market, e.g., mobile Internet services for personal users through 5G smartphones and fixed wireless access (FWA) devices. Along with the rapidly evolving 5G technology, there has been an increase in 5G-enabled innovations in vertical sectors like industrial automation, healthcare, and public safety [1]–[4].

In the healthcare sector, medical device innovators are exploring the incorporation of 5G connectivity in their product development roadmaps [5], [6], [8], where 5G is envisioned to augment how medical devices communicate with each other and with external resources and to enable novel connected medical applications. In a recent landscape analysis of 5G in healthcare published by the Medical Device Innovation Consortium (MDIC), a large group of stake-holders including medical device manufacturers, healthcare providers, telecommunication vendors, major MNOs, as well as government agencies, has shared a broad vision of 5G-enabled medical device applications like medical extended reality (MXR), mobile medical units, robotic assistance in remote surgery, and telemedicine [3]. The document also identifies knowledge gaps and barriers that may hinder the safe delivery of 5G benefits in healthcare to patients.

Among the knowledge gaps is the lack of evaluation methods that can be used to accurately assess the role of 5G connectivity in supporting the performance of medical devices [3], [9]. Developing an evaluation method in this space is complicated by opaque 5G network implementations, a lack of understanding and documentation of connectivity failure modes that can be mapped to practical use-cases, and unclear integration practices [9]. Notably, documenting the role of the adopted connectivity modality in enabling a medical device function and its impact on the device safety and effectiveness is a component of the device premarket submission to the U.S. Food and Drug Administration (FDA) [11] before the device can be legally brought to market. Therefore, innovative medical devices adopting emerging technologies like 5G need evaluation tools (e.g., test beds, test protocols, models) to inform their benefit-risk assessment when they are considered by FDA [10].

**FIGURE 1:**
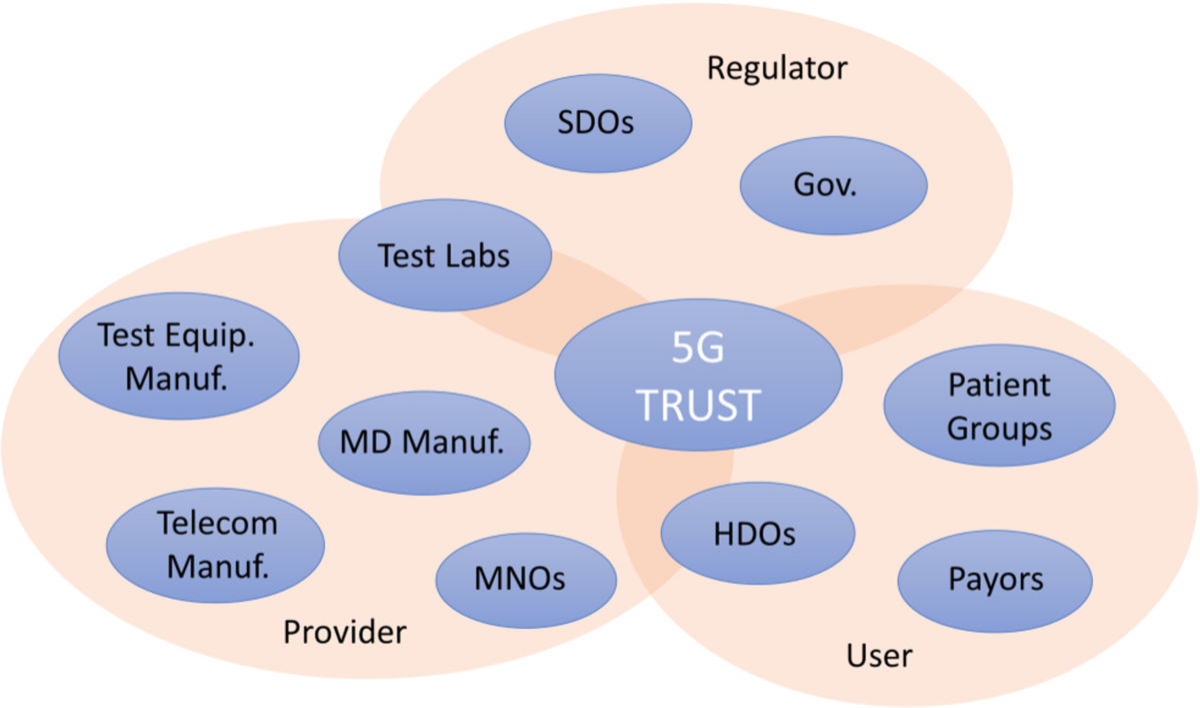
TRUST stakeholders including several categories of manufacturers (manuf.), standard development organizations (SDOs), health delivery organizations (HDOs), and government agencies (gov.).

In this paper we propose a new testbed design model named Testbed as a RegUlatory Science Tool (TRUST). The TRUST model is focused on assessing the role and impact of using 5G connectivity for enabling medical device functions, which can be a valuable tool for many stakeholders in the connected medical device ecosystem as illustrated in Fig. 1. While those stakeholders might have different motivations for wanting to evaluate the use of 5G in medical devices, they converge around the need for a simple and flexible testbed design to perform this evaluation. We first identify essential network components and features for implementing 5G systems and over-the-top medical services in a testbed. We formalize the model infrastructure by organizing different testbed functions in separate but cross-linked panels, each with specific key features and elements. We also discuss the design principles in selecting and implementing the individual panel components to facilitate a least-burdensome evaluation.

With the lack of voluntary consensus standards for evaluating 5G-enabled medical devices, innovators in this space are left to devise their own ad-hoc testbeds and evaluation methods. The work detailed in this paper is a step toward facilitating an evaluation approach that can accommodate the diverse evaluation needs in this domain. The contributions of this paper are three-fold: first, we identify the dual roles of 5G-enabled medical devices, i.e., being medical equipment and 5G user equipment (ME-UE), with unique evaluation needs for each; second, we propose a new testbed design model, i.e., TRUST, that details design considerations in testbed organization, implementation options, and customization per test objective; lastly, we verify the feasibility and flexibility of the TRUST model in a realistic 5G testbed implementation in the Wireless Laboratory of the Office of Science and Engineering Laboratories (OSEL), Center for Devices and Radiological Health (CDRH), FDA. A practical test process is demonstrated through a 5G-enabled MXR application example.

Table 1 lists the abbreviations used in this paper. The remainder of this paper is organized as follows. Section II overviews related work at the intersection of medical device and 5G test tools. Section III provides a background on regulatory science tools and the motivation for the TRUST design. Section IV discusses the main infrastructure and design principles of TRUST. Section V reports on a realistic example for using TRUST for testbed development. Implementation challenges are discussed in Section VI. Section VII concludes the paper.

## II. RELATED WORK

5G-enabled medical devices are UE nodes in the 5G system and as such they—or the 5G chipsets they contain—undergo certification testing before they are admitted to a live 5G network. These tests address regulatory requirements for spectrum access, industry group standards, and carrier specific requirements in the target deployment market. The report in [9] reviews the tiered certification testing architecture and outlines its limitations in supporting the safety and effectiveness of medical devices. A part of those certification tests is published by the 3^rd^ generation partnership project (3GPP) new radio (NR) releases as the *de facto* specification for 5G systems, which include a set of test methods and recommended test cases for UE devices, radio access network (RAN), and core function elements [14] and are widely referenced by developers and standard development organization (SDOs).

Experimental trials were reported that verified medical device applications over real 5G network connections with technical assistance from 5G equipment vendors and MNOs [5], [6]. However, leveraging real carrier networks for testing and evaluation might be impractical for incorporation in iterative device development cycles. Similar to the evaluation practices in the other complex systems, e.g., power grids, transportation system, and the Internet, 5G testing also heavily utilizes system models and empirical data to simulate real system behaviors and environmental factors. 5G network simulators were developed for academic research and technical verification purposes, e.g., 5G LENA in NS3 [15] and Simu5G package in OMNET++ [16]. These programs adopt evolving 5G channel models whose development is based on-site measurement campaigns in representative topographies [17], [18]. The introduction of software-defined radios (SDR) with flexible RF configurations and programmable transceiver (TRX) behaviors has permitted 5G tests in programmable hardware assemblies [19]. Meanwhile, open-source software projects also contributed to making 5G network functions and protocol stacks open for research and third-party development, e.g., srsRAN [20], OAI [21], and open5GS [22]. Enabled by these techniques, evaluating 5G system services using testbed platforms has become a feasible, and in many cases cost-effective, test approach.

Small- and large-scale wireless testbeds were developed to operate shared and reusable testbed facilities for experimenting with emerging wireless techniques including 5G systems and applications, e.g., the platforms for advanced wireless research (PAWR) programs funded by the National Science Foundation (NSF) [23]. These tools have equipped wireless researchers and device developers with the capability of defining, building, and accessing self-managed and private 5G networks where they can verify UE product designs and innovations with near-realistic setup ahead of real network trials. Notably, there are efforts for coordinating distributed testbed resources to form a shared pool [24].

5G-enabled medical device applications are diverse in their connectivity needs and so are the 5G network deployment options that can be adopted by service providers. Accordingly, a one-size-fits-all testbed approach is impractical and might result in a mismatch between the medical device evaluation needs and testbed capabilities. Therefore, TRUST is focused on identifying the essential 5G system elements to create flexible testbeds for evaluating medical device connectivity.

## III. REGULATORY SCIENCE TOOLS

A regulatory science tool (RST) is an innovative, science-based approach or methodology to help assess the safety or effectiveness of a medical device or emerging technology. Examples include phantoms, test methods, testbeds, computer models, data sets, and recommended practices. Developing RSTs can be considered an agile approach for rapidly bridging knowledge gaps in the development and evaluation of medical devices, which can facilitate innovation while promoting medical device safety and efficacy and reduce the need for ad-hoc device testing [10]. A catalog of RSTs addressing many medical device scientific domains (e.g., bio-compatibility, imaging, artificial intelligence, wireless technology) is publicly available by FDA/CDRH/OSEL [31].

Many medical devices use wireless technology to deliver their intended function. The FDA guidance document on RF wireless technology in medical devices [11] discusses considerations that can help support the medical device safety and effectiveness. Examples include the selection of wireless technology, quality of service, coexistence, security, and electromagnetic compatibility (EMC). Notably, emerging topics in wireless technology use in medical devices are often not addressed in voluntary consensus standards. For example, the guidance document provided timely recommendations for addressing the emerging issue of wireless coexistence, but there were no standardized test methods available at the time. As a result, medical device manufacturers and test labs continued to use their own ad-hoc approaches that inconsistently addressed the issue, which increased burden for industry and FDA regulatory reviewers. Given the lack of standards, RSTs were developed to bridge the gap and equip both industry and the FDA with preliminary evaluation methods [25]–[27]. These RSTs informed the development of, and were later integrated in, standardized documents for the risk management and evaluation of wireless coexistence [13], [30].

**FIGURE 2:**
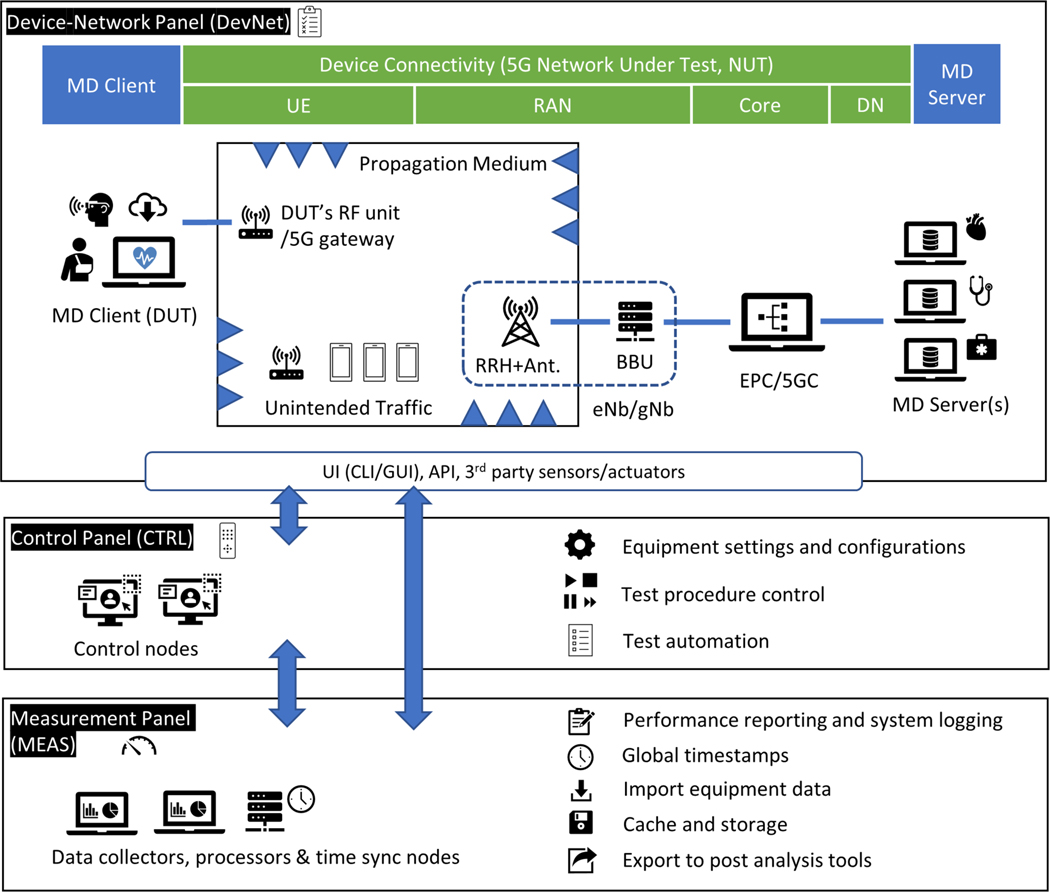
TRUST design model for testbed function panels for evaluating 5G-enabled medical devices. Medical device is abbreviated by MD, data network is abbreviated by DN, remote radio head by RRH, antenna by Ant., baseband unit by BBU, 4G evolved packet core by EPC, 5G core by 5GC, command line interface by CLI.

As an emerging connectivity modality in healthcare, the challenges for evaluating 5G use in medical devices are similar to those wireless coexistence used to face, e.g., there are currently no standardized methods to perform this evaluation. As discussed in [9], existing 5G test methods are limited in supporting the safety and effectiveness of medical devices. RSTs in the field of 5G-enabled medical devices should a) enable device innovators to focus their effort on developing their products instead of designing testbeds and artifacts; b) recognize the diversity and unique characteristics of connected medical applications and hence the need for customizable test solutions; and c) include detailed instructions for use to rapidly deploy and obtain desired outcomes. TRUST attempts to achieve those objectives as a step toward facilitating innovation and supporting the development and evaluation of safe and effective connected medical devices as detailed in the remainder of this article including in Fig. 2, which illustrates the TRUST design model using function panels, Table 3, which suggests example implementation profiles based on the desired testbed use case, and Protocol 1, which includes a complete example for implementing and operating a use case using the TRUST design model.

## IV. TESTBED AS A REGULATORY SCIENCE TOOL (TRUST) DESIGN FRAMEWORK

Among the diverse options for evaluating 5G-enabled medical devices, e.g., theoretical models, network simulators, and trials in real networks, testbeds offer a flexible and detailed approach for depicting the 5G networks use by medical devices under test (DUT). On one hand, testbeds can employ realistic equipment and environment elements, e.g., 5G channels, to replicate realistic use cases. On the other hand, they are also able to provide cost-effective solutions by integrating simulated or emulated components, where relevant, to meet a desired balance between modeling accuracy and precision.

The TRUST model adopts a modular design for organizing various testbed functions, implementing testbed components, and categorizing 5G connections under test according to the test objectives. In the remaining part of this section, we will elaborate on the detailed TRUST design.

### A. FUNCTION PANELS

Fig. 2 illustrates the overall TRUST architecture for designing 5G testbeds. A TRUST testbed consists of three function panels, each consisting of unique components: device-network (*DevNet*), control (*CTRL*), and measurement (*MEAS*). The panel-based structure provides an open design blueprint for device developers and testers. In this context, device developers can leverage TRUST to focus on the characterization of the DUT, e.g., connectivity requirements of the device functions carried by 5G, quality of service (QoS) criteria, identification and design of control and monitoring interfaces. The bidirectional flow of information between the different testbed panels is illustrated with the blue double arrows in Fig. 2. Testers can benefit from a straightforward process for standing up a testbed that meets the DUT testing needs and permits transparent observations to be documented.

#### DevNet Panel

The DevNet Panel consists of the medical DUT and the 5G network under test (NUT). The former is the subject device system including the user-end hardware/software (i.e.,client application) and the remote hardware/software (i.e., server application). The latter creates the 5G connectivity that enables the DUT communication in its intended work mode. According to the testing purpose, unique sets of network elements may be deployed in the NUT to create active 5G connections as well as stress conditions, e.g., background traffic and interference signals. The solid blue lines connecting various components of the DevNet Panel in Fig. 2 refer to the interconnections between network elements. Depending on the desired testbed implementation options, these connections might be made using physical media like wires and fiber optic cables or logical links in case simulators and emulators were used.

#### CTRL Panel

The CTRL Panel aggregates the control functions of DevNet Panel objects, e.g., the equipment settings and configurations including the sensors and actuators that might help replicate realistic use cases or manage test procedures. It includes the lab equipment used to manage (i.e., configure, monitor, and maintain) the testing environment. Test automation, e.g., start/pause/stop/reset the application/network, can be realized by utilizing available user interfaces (UI) and application programming interfaces (API) to promote accuracy and precision in iterative test cycles.

#### MEAS Panel

The MEAS Panel collects and stores test data from the DUT and NUT including the application status, network traffic, and equipment diagnostics. It also archives the configurations files used in the CTRL Panel. The stored data will be used during post analysis to document the test observations and outcome. Another important function of this panel is to synchronize distributed nodes in the testbed so that the collected data have a timestamp that is synced to the same time source. The MEAS Panel may also coordinate with the CTRL Panel for recording test controls and inputs as a part of the test data to correlate DUT responses to specific test vectors.

TRUST adopts a modular design for organizing testbed elements and functions. The following are notable features of this design. First, TRUST promotes the adoption of standardized interfaces between function modules so that developers or testing labs can replicate the design with equipment they already have, flexibly acquire new equipment, or develop their own implementations. For example, the NUT in the DevNet Panel represents 5G system modules and interfaces per 3GPP technical specifications. The modules along the 5G data path, e.g, UE or RAN entities, can use commercial products or simulated/emulated entities to meet the evaluation objectives and constraints granted that the replacement supports the same physical media (wireless or wired) and protocol stack.

Second, TRUST facilitates the vertical integration of test equipment across function panels. One physical instrument in the testbed can play multiple roles in different panels. For example, a 5G base station serves as the RAN facility of the NUT in the DevNet Panel and the server module of a control graphical user interface (GUI), e.g., Nokia AirScale Web Element Manager (WebEM), in the CTRL Panel, which is also the information source for RAN traffic captures in the MEAS Panel.

Third, the modular design permits future upgrades as an open design framework. Each part can be replaced with a more advanced or novel solution so that the evaluation platform has a longer service lifetime compared to fixed setups serving specific one-time testing needs. For instance, the testbed can evolve to verify new 5G features, e.g., a new Core network function in a new 3GPP release, by adding corresponding NUT functions or system elements in DevNet Panel. Meanwhile, TRUST would also facilitate adding or updating the corresponding interfaces and functions in CTRL and MEAS Panels. These changes across the panels will jointly serve as a complete solution for evaluating the evolving DUT connectivity needs.

### B. 5G NUT ENTITIES AND IMPLEMENTATIONS

A representative 5G NUT includes an end-to-end network path connecting end users to the remote application services, which is implemented in the DevNet Panel. TRUST identifies the following NUT entities as element containers that host corresponding 5G functions along the complete path: *UE*, *5G channel*, *RAN*, *Core*, and *Data Network (DN)*.

As shown in Fig. 2, UE is considered the user-end portal to the 5G network for medical devices. This is true whether the device contains a 5G chipset or uses an external gateway device for network access. The physical media of 5G channels are also considered as an independent NUT entity in TRUST since the testbed design involves the realization of wireless data transmissions, where the propagation medium can impact the connectivity key performance indicators (KPIs) like data rate, latency, and reliability. RAN consists of cellular base stations, i.e., 4G eNodeB (eNB, for non-standalone mode [NSA]) and 5G gNodeB (gNB, for NSA/standalone [SA]). UEs and RAN communicate with each other through 5G channel, that can be realized using a conducted propagation path or over-the-air in shielded enclosures or open test ranges. The choice of channel media follows the desired implementation fidelity compared to realistic networks and the availability of test facilities (e.g., anechoic chamber) and test spectrum licenses for operating in open spaces. RAN serves at the edge of the 5G network facility that is commonly operated by MNOs. RAN elements are connected to Core that acts as the MNO’s backbone managing all user and RAN profiles and providing access to remote applications. The portal at Core to DN can be considered as the perimeter of the 3GPP NR system. Accordingly, DN is any external Internet service and its network infrastructure that provide over-the-top mobile applications to 5G users including medical devices. TRUST includes DN so that the testbed can establish a clear interface allowing the DUT to integrate their remote application, e.g., server-side component or the companion device, into the NUT.

The implementation options for NUT entities can be classified in three types: real case (R) applications, emulation (E) by hardware, and software simulations (S). Table 2 summarizes the advantages (indicated by the + sign) and disadvantages (indicated by the - sign) of the implementation options for each 5G NUT component. The stated characteristics are considered in abstract and do not address specific test cases. Comparisons are made between approaches assuming similar network scale. Notably, among multiple options for the same component, the resolution of depicting system behaviors increases from S to R, the same as the cost estimate, while the implementation flexibility decreases. Although a fully simulated 5G NUT can be beneficial to researchers and developers exploring novel device functions, the mathematical models and empirical data referenced by the simulators obscure many operational details that might help inform the benefit-risk analysis of a 5G-enabled medical device by documenting its expected real-world behavior. Realistic implementations can be desirable to help observe the realistic system performance and failure modes. However, their use is sometimes impractical. For example, early in the device development cycle when the device connectivity needs are still evolving and the desired communication features are being explored. In this scenario, an E/S option might offer a more flexible and cost-effective solution for meeting the device evolving evaluation needs. Notably, the system complexity and integration difficulty increase from UE to central control entities in the 5G network. Implementing multiple UE nodes (e.g., smartphones) in a testbed is relatively straightforward. Deploying carrier-grade RAN and core equipment is a more involved process that is often performed by specialized vendor integration teams. Accordingly, testbeds might adopt an approach that mixes R/E/S components to address the desired evaluation objectives.

### C. EVALUATION-ORIENTED TESTBED DESIGN

The unique connectivity needs of different 5G-enabled medical devices and the stage of development for a particular device can inform the desired technical capabilities in a 5G testbed, e.g., enabling features, setup configurations, performance probing capabilities, etc. While Table 2 referenced practical tradeoffs for selecting testbed components that are simulated, emulated, or realistic, Table 3 lists example scenarios for the evaluation objective, how they can be implemented according to literature reports, and how the implementation is mapped to the TRUST model. The purpose of this table is to showcase how test needs influence a testbed end design and implementation. The scenarios can be categorized as follows.

*Cat. 1* tests are focused on medical device functions that are enabled by 5G connectivity. Accordingly, the 5G connectivity setup involves the end-to-end 5G NUT path that interfaces with the real DUT and its remote companion entity. Medical devices might leverage a gateway to access the 5G network as illustrated in Cat 1.1 or use embedded 5G radio modules like the case of Cat 1.2. Cat 1.3 captures the replication of a device field trial in its use environment leveraging commercial 5G service, which can be used to support MNO UE acceptance tests [9].

*Cat. 2* tests are focused on the communication path between UE and DN. These tests can be applicable to the development and verification of the 5G radio module design for medical devices or focusing on specific connectivity failure modes that are relevant to the device. Basic network operations are included in these tests, e.g., UE communications with core network functions (through RAN) such as message exchanges in the S1AP protocol (for 5G NSA Option 3x) and NGAP protocol (SA NR Option 2) for UE attach/detach processes. The tests examine the 5G protocol stack implementations and their conformance with particular 3GPP releases. Table 3 lists six example tests in Cat. 2.1 to 2.6. The use of simulation permits the efficient examination of specific and limited features or algorithms like in Cat 2.6. On the other hand, UE conformance and acceptance tests adopt realistic and emulated implementations.

*Cat. 3* tests are focused on the physical (PHY) link performance, which is commonly limited to 5G transceiver pairs. Cat. 3.1 represents tests similar to the Federal Communication Commission (FCC) emission tests for assessing the UE emissions at the maximum power level in alignment with the relevant licensed band limits [32]. In addition to verifying 5G PHY design, the setup per Cat. 3.2 can also be used to generate RF emissions as unintended signals used for interference testing. Cat. 3.3 can be used to study the RF link through simulation, which can be done without the need to simulate core or data network elements.

## V. A TRUST IMPLEMENTATION IN THE 5G TESTBED AT FDA

As a practical use of the TRUST design model introduced in Section IV, we detail hereafter the development of a 5G testbed at FDA/CDRH/OSEL, Silver Spring, Maryland. The testbed serves as a platform for developing evaluation methods of the connectivity enablers of 5G medical devices and allows the identification and control of key 5G system components for assessing 5G connectivity. As a practical implementation, the testbed has limitations and design tradeoffs that are discussed in the following sections.

### A. 5G NUT ENTITIES

Table 4 provides an overview of 5G NUT entities implemented in the testbed’s DevNet Panel. Specifically, we adopted a mixed technical path as discussed in Section IV-A, which consists of realistic systems and emulators. The former were deployed for the UE, 5G channel, and RAN components; the latter addressed the core emulators and air-gapped servers in DN. This selection highlights a trade-off between modeling accuracy to characterize the system behavior in over-the-air and RAN aspects of the connection and operational feasibility for the core and DN (e.g., function availability, performance traceability, and cost).

Two 5G network architectures are supported: NSA E-UTRAN New Radio – Dual Connectivity (EN-DC) Option 3x and SA NR Option 2. The former leverages existing RAN and Core deployed in a 4G systems to add new 5G RAN capabilities. Accordingly, it has been widely used by MNOs during the transition from 4G to 5G. The latter provides a native 5G network solution in both the RAN and Core.

A UE node is used as a 5G gateway (UE-GW) for connecting a DUT to the 5G NUT. As detailed in Table 4, a carrier-grade 4G/5G Nokia base station (BTS) is deployed in the RAN that comprises baseband units (BBU) and remote radio heads (RRH) for 4G eNB and 5G gNB. The BTS works with a Valid8 core emulator that runs on a customized Linux server. The 5G NUT can alternate between NSA and SA modes by loading the corresponding configuration files to set up the RAN and core entities. RF cells can be initiated in three frequency bands. In frequency range 1 (FR1, also known as sub-6 GHz), the eNB in NSA mode runs in B66 and the gNB in NSA/SA modes runs in N2. In FR2 (also known as mmWave), the gNB in NSA mode runs in N260.

An anechoic chamber is used for enabling wireless OTA experiments. The 5G channel is formed by placing UEs and RRHs in the chamber where they are shielded from commercial cellular transmissions in the vicinity. The setup also ensures that transmissions from the testbed do not interfere with licensed networks. The DN entities include common services such as IP Multimedia Subsystem (IMS) for voice and video calling and IP connection testing suites (e.g., iPerf3).

The testbed is operated in a “sandbox” environment whose hardware, software, and operations are not only isolated from commercial telecommunication activities but also from internal enterprise IT networks. While not implemented in this testbed, we recognize that connectivity with external networks might be needed in some cases like the DUT’s remote application relying on service engines on cloud platforms such as Amazon Web Services (AWS) or Microsoft Azure. Interested readers can learn more about cloud-based testbed options by referring to the Federal Mobility Group white paper in [24].

### B. A 5G MXR DUT SETUP EXAMPLE

We introduced an example medical extended reality (MXR) application as the DUT in the testbed. MXR applications implement augmented reality (AR) and/or virtual reality (VR) technologies providing an immersive user experience with virtual objects and/or environments through head-mounted display (HMD) devices. Emerging MXR device applications are received through FDA’s premarket medical device submissions in areas like telemedicine with remote physicians, virtual surgical planning, and patient rehabilitation [7], [33]. MXR applications also have many evaluation challenges as detailed in [33]. Existing HMD devices and MXR development platforms commonly use wired/wireless local access networks (LAN) as network solutions connecting MXR users to application servers. However, 5G has been recognized as a connectivity modality to enable MXR applications with high-throughput, low-latency data services along with enhanced mobility and security features [3], [28].

**Protocol 1:** 5G MXR Test Case Demo

**FIGURE 3:**
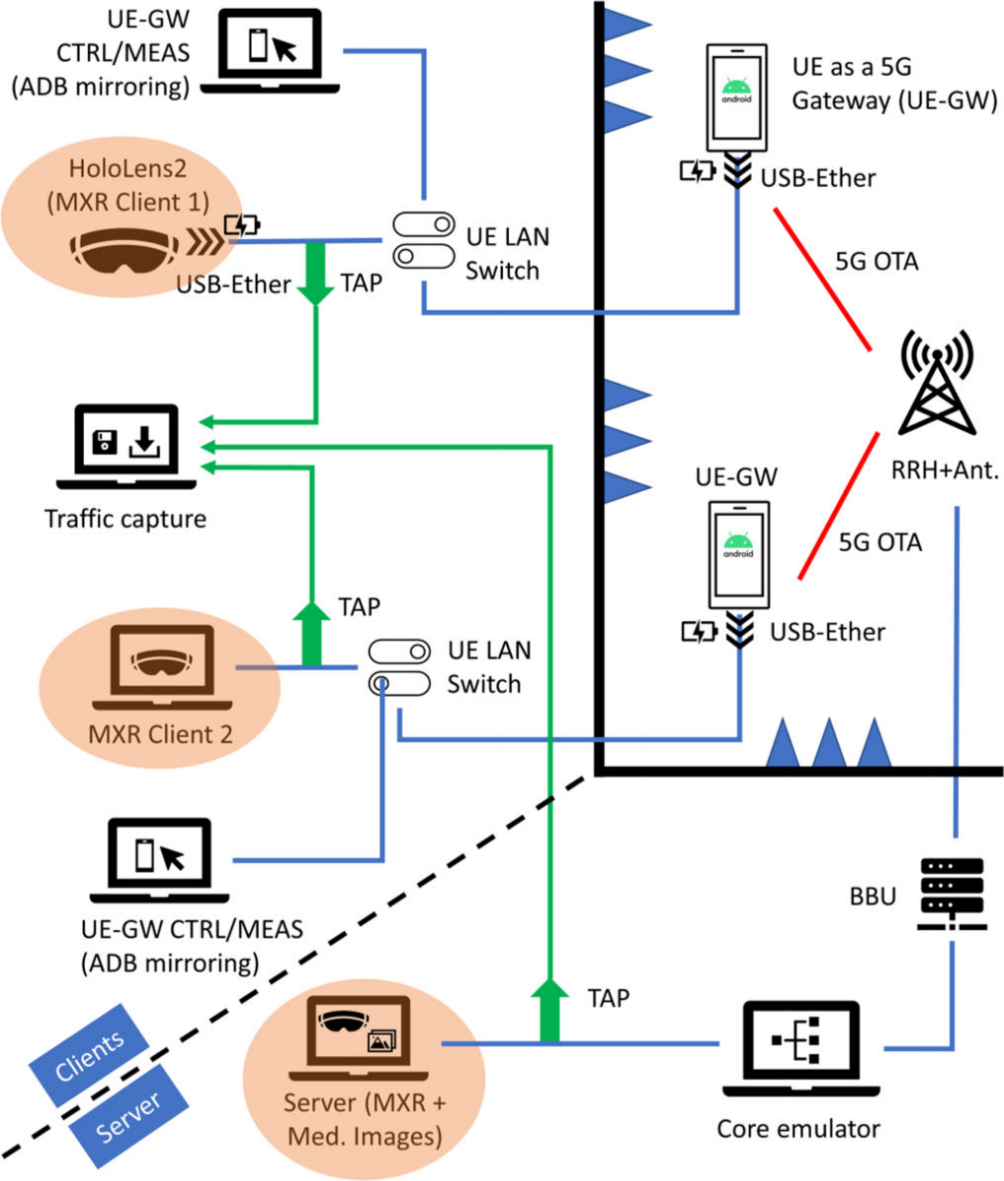
MXR device setup and data collection

*Inputs.* 5G NUT configuration files per Table 4, other panels configuration files per Table 5.
*Goal.* The MXR server and clients communicate over 5G NSA connections.
*Test protocol:*
1) **Setup (DevNet/MEAS).**
a) One server and two clients of the DUT are connected to the 5G NUT per Fig. 3.
b) 5G NUT operates in tri-band (B66/N2/N260) NSA.
c) TAP devices sniffing DUT traffic are connected to the collector in MEAS Panel per Fig. 3.
2) **Procedure (CTRL/MEAS).**
a) Start 5G NUT.
b) Verify 5G UE connections and test round-trip time (RTT) and throughput (TCP/UDP) in connected links.
c) Start MEAS Panel link traffic recording.
d) Start MXR players (server and clients) in the order (Server, HL2, Win.Cli.).
e) Verify MXR client-server connections.
f) MXR players request medical images on the virtual display in the order (Server, HL2, Win.Cli.). Repeat for three rounds.
g) Change Win.Cli.’s field of view
h) Stop MXR players (server and clients) in the order (Win.Cli.-HL2-Server).
i) Stop MEAS Panel data recording.
3) **Output (CTRL/MEAS).**
a) NUT performance log files.
b) 5G UE connection test results.
c) TAP captures for MXR player traffic.

The MXR DUT in this demo was a custom-built multi-user virtual clinical room with CT image display capability. The application was built using the Unreal Engine platform, which has been widely used for extended reality (XR) applications. The MXR application server (“server”) creates a virtual MXR environment (“virtual map”) capable of hosting multiple MXR application users (“clients”). The clients and the server are represented by their 3D avatars in the virtual map mimicking their real-world movements and gestures that were captured by their HMD devices using built-in sensors and cameras. Each client reports their state changes to the server and receive updates of the other clients during a client-server session. In addition, the application also provides access to a medical image library that was deployed at an HTTPS multimedia server. Every participant, i.e., the client or server, could request to download and display a new image in the virtual map that is distributed to all participants’ views by the server. The tools used in the DUT development are listed in Table 5, which also summarizes CTRL and MEAS panel functions that correspond to the selected NUT entities. Some of these functions are native to the used equipment like controlling an RF cell using ON/OFF switches. Others were developed using open-source software tools on general computing platforms, e.g., UE monitoring and IP traffic sniffing. The TRUST model focuses on how the different testbed panels work and interact to enable a test platform, which provides a reference for stakeholders interested in developing their own evaluation platforms.

To enable the MXR DUT connectivity, the testbed was configured according to the profile serving functional testing for 5G MDs, i.e., Cat. 1.1 in Table 3. Specifically, we deployed two MXR clients, i.e., one Microsoft Hololens 2 (HL2) HMD and one Windows computer client (Win.Cli.), both equipped with software for joining the multi-user test. Each used a separate 5G UE device, i.e., Samsung Galaxy S21 Ultra, as the gateway to the 5G NUT. The MXR server and the image server were installed on the same Windows computer deployed in the testbed DN. The client-server communications in the MXR sessions were carried by 5G RF links. Fig. 3 illustrates the network setup at the client and server ends as well as CTRL and MEAS panel configurations per Table 5. The design details of the MXR demo and test procedure will be the subject of a future publication. Protocol 1 details the test procedure for documenting the end-to-end 5G-MXR connectivity as a comprehensive example for incorporating a DUT in a TRUST testbed.

### C. TESTBED DATA ANALYSIS

This section presents three sets of testbed data collected to demonstrate diverse evaluation tasks.

#### 1) System Function Check

The first data set focuses on mmWave beamforming as an example of evaluating specific 5G system features. Beamforming is a physical layer technique used in 5G channels to focus the signal emissions/receptions at a multi-input multi-output (MIMO) antenna array toward/from a narrow direction or area, also referred to as a “beam”. It is commonly used to enhance the link quality in 5G channels, especially in the FR2 bands to mitigate the high signal attenuation at mmWave frequencies.

**FIGURE 4:**
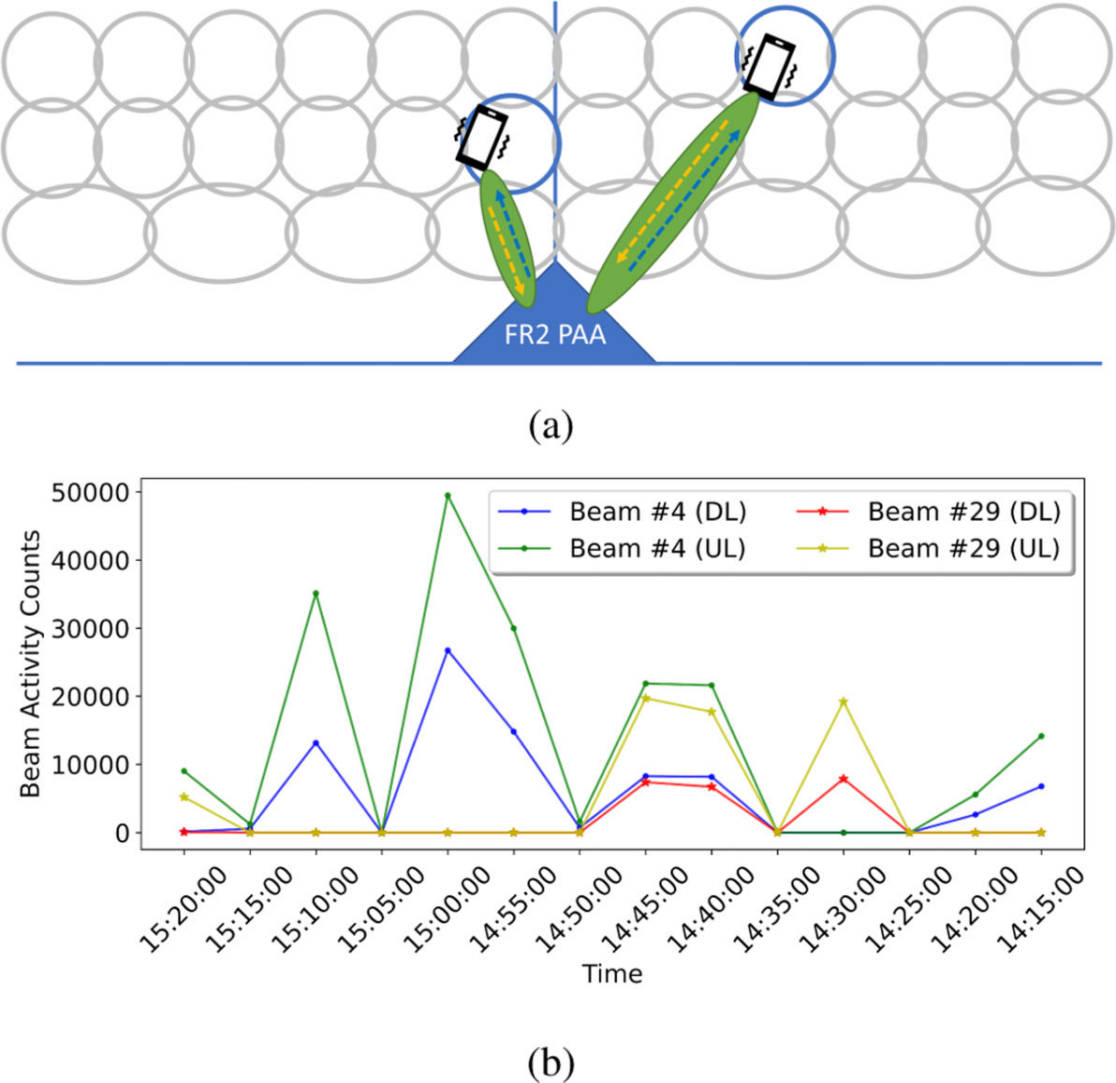
Beamforming in the mmWave cell. a) illustration of beams in use, b) active beam usage counts per RAN logs.

The beamforming function was activated in the mmWave cell operating in NR band n260 at 39 GHz. The cell covered 180-degree area served by one Nokia AirScale AEWD mmWave radio, which divided the cell into 32 beams as illustrated in Fig. 4a. The mmWave radio comprises two phased antenna array (PAA) panels, each serving a 90-degree area. The beam set configuration was “6_6_4”, i.e., each FR2 radio panel served 16 beams that were aligned in three rows with 6, 6, and 4 beams per row, respectively. The output was performance indicators captured by the Nokia 5G BTS management tool WebEM.

UE-GW smartphones were placed at stationary and unique spots in the chamber as shown in Fig. 4a. Two beams in the mmWave cell, i.e., Beam ID 4 and 29, were observed with active downlink (DL, i.e., from RAN to UE) and uplink (UL, i.e., from UE to RAN) usage during the test as shown in Fig. 4b. The y-axis reports a gNB performance indicator that counts the times the corresponding ID of an active beam was recorded in the DL and UL physical channels in each 5-minute reporting interval. The higher the value, the more frequent the beam was used for data transmissions. The x-axis is the time range of a measurement campaign consisting of a sequence of independent test rounds that were separated by short breaks for test preparation and conclusion tasks. Notably, the beam activities varied with time depending on the UE activities enabled in different test cases where either one or two 5G links were exercised with traffic load.

#### 2) NUT Link Verification

This data set reports on link measurements collected to verify the state of the testbed and ensure readiness for incorporating a DUT. For example, a step in Protocol 1 was to verify the 5G links before enabling the MXR application. The link verification can be divided into three sub-steps including 1) UE attach check, 2) IP ping tests, and 3) link performance tests.

Specifically, the attach process of each 5G UE-GW was verified by inspecting the S1AP protocol handshakes in the interface between the RAN and the Core, which included equipment capability checks and configuration negotiations during a UE attach. A successful UE attach could be noted by the session confirmation message sent by the Core that leads to displaying a “5G” icon on the top of the smartphone’s screen. Next, IP ping test was used to verify if the NUT was successfully connecting the attached UE and the DN server. The server IP was pinged from the UE and the results recorded, which also provided the latency information in term of the end-to-end round trip time (RTT) as shown in Table 6. The following step was to measure the throughput performance in the 5G connection by running iPerf3 with TCP and UDP traffics. Table 6 summarizes the measured performance.

#### 3) 5G DUT Characterization

The application traffic was captured at individual DUT nodes, i.e., the server and the two clients, as shown in Fig. 3. Network test access point (TAP) devices were inserted into the access links from terminal nodes and copied the through traffic flows to a central point; the machine at that point was operating multiple Wireshark instances, one for each TAP, to record inbound and outbound messages at individual DUT nodes. An advantage of this setup is that the timestamps of captured packets were all obtained from the same clock, i.e., the collector, so that the messaging timeline can be accurately analyzed. Notably, assessing the medical function of the 5G DUT is beyond the scope of this report.

**FIGURE 5:**
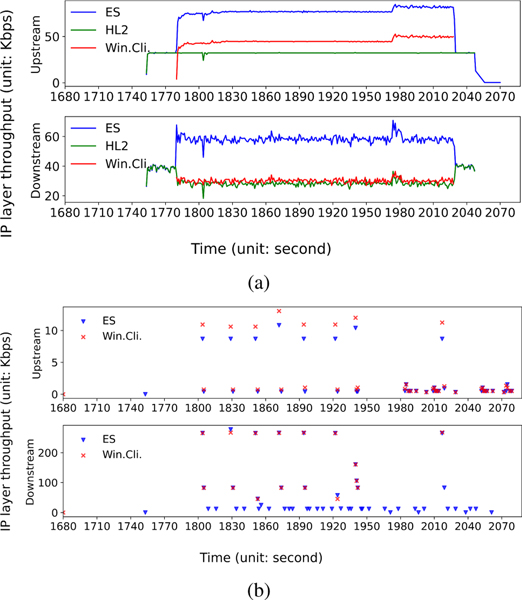
Dual client scenario IP layer throughput per Node versus time (a) UDP (b) TCP. ES refers to Edge Server, which hosts the MXR server application. HL2 and Win.Cli. refer to HoloLens 2 and Windows client, respectively.

Fig. 5a and Fig. 5b illustrate the changes in IP layer packet throughput as observed through UDP and TCP streams during testing at individual DUT nodes. The example MXR client and server routinely communicated through UDP packets for updating node and environment states and remote procedure calls (RPCs). Each node downloaded a medical image when a new image request was initiated, which was included in an HTTPS session through TCP. The results do not show TCP download traffic at the HL2 node because the HL2 tended to cache image files locally after they are first received. This feature in the Unreal engine libraries can improve response time in connected XR applications. However, the same behavior was not observed in the Windows client and TCP traffic was documented and associated with each image request.

**FIGURE 6:**
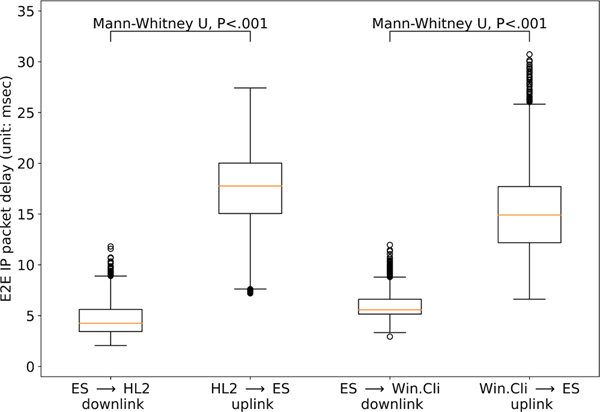
The end-to-end delay performance of the DUT’s UDP flows. Samples collected in the downlink and uplink were compared through Mann-Whitney U tests with p values less than 0.001.

The end-to-end transmission latency of the DUT’s routine update messages in UDP is illustrated in Fig. 6, which indicates that client-initiated UDP messages generally experienced a longer delay in the transmissions compared to the ones initiated by the server. Such an observation was obtained through Mann-Whitney U tests on the downlink and uplink delay samples where p values were less than 0.001. This observation highlights that latency considerations in 5G-enabled medical devices should be understood on both the uplink and downlink since a lack of support for certain latency requirements in either direction might impact the connected device intended function.

## VI. IMPLEMENTATION CHALLENGES

The TRUST model specifies the NUT components and interfaces between them, which facilitate discussions about the development and implementation of a 5G testbed with internal and external stakeholders. This is particularly relevant if connecting the testbed to external networks (e.g., Internet, other testbeds) is part of the test plan. Accordingly, communicating and coordinating with an organization’s networking team to facilitate this capability can be done efficiently. However, integrating a testbed within an enterprise network is not straightforward especially when factoring the organization’s policies for data privacy and security.

Another challenge is the interoperability of network equipment across vendors and even multiple system versions of the same vendor. Although 3GPP specifications detail 5G features, practical implementations by equipment vendors often result in the need for additional effort when integrating network elements to troubleshoot integration errors and resolve inconsistencies. There is a significant body of work in the area of network interoperability to address this challenge. For example, OpenRAN promotes the interoperability of multi-vendor products [46].

5G testbeds are more complex constructs compared to testbeds addressing other wireless technologies commonly used in medical devices like Wi-Fi and Bluetooth, where evaluating wireless coexistence is commonly the focus of testing [47]. There are consensus standards that detail the wireless coexistence test setup and specify test vectors (e.g., unintended signal amplitude, channel occupancy, modulation and coding scheme) that are presented in increasing evaluation thoroughness following the risk category of the medical device wireless function [13], [30]. To the best of the authors knowledge, no similar standards exist for 5G. Compared to Wi-Fi, 5G includes significantly more network elements to establish end-to-end connectivity. Therefore, simple tasks related to evaluating connectivity become more complex to realize. For example, the IP addresses of 5G UE nodes often change between attachments, which makes it more cumbersome to ping a specific UE node compared to pinging a static IP address associated with a specific LAN node.

## VII. CONCLUSION

We introduced the TRUST model for developing a 5G testbed to facilitate the evaluation of 5G-enabled medical devices. Accordingly, it addresses the end-to-end communication paths and how to implement them using real, emulated, or simulated components. A practical testbed implementation using TRUST was detailed along with an example 5G-enabled medical device application that demonstrates the incorporation of a DUT in the testbed and the diverse data that can be collected.

Future work will include enriching the testbed capability with evolving 5G system features and developing evaluation methods for addressing connectivity failure modes that might impact connected devices use. Accordingly, the testbed and evaluation methods will contribute to the innovation cycle by helping innovators efficiently evaluate their emerging products, equipping regulators with an approach to assess the validity of submitted evaluation data, and facilitating patient access to innovative, safe, and effective connected medical devices.

## Figures and Tables

**TABLE 1:** List of Abbreviations

Abbrev.	Meaning

3GPP	3^rd^ generation partnership project
5G	Fifth-generation (mobile communication)
5GC	5G core
API	Application programming interface
AR	Augmented reality
BBU	Baseband unit
BGP	Border Gateway Protocol
BTS	Base station
CDRH	Center for Devices and Radiological Health
CLI	Command Line Interface
CT	Computed tomography
CTRL	Control
DevNet	Device-Network
DL	Downlink (from RAN to UE)
DN	Data Network
DUT	Device under test
E2E	End-to-End
EMC	Electromagnetic compatibility
eNb	eNodeB (4G base station per 3GPP)
EN-DC	E-UTRAN New Radio - Dual Connectivity
EPC	Enhanced packet core
FCC	Federal Communication Commission
FDA	Food and Drug Administration
FR1(2)	Frequency range 1(2)
FWA	Fixed wireless access
gNb	gNodeB (5G base station per 3GPP)
GUI	Graphic User Interface
HDO	Health delivery organization
HL2	Hololens2 (HMD device)
HMD	Head-mounted display
HW	Hardware
IMS	IP multimedia subsystem
ISP	Internet service provider
KPI	Key performance indicator
LAN	Local area network
MD	Medical device
MDIC	Medical Device Innovation Consortium
MEAS	Measurement
ME-UE	Medical equipment and (cellular) user equipment
MIMO	Multiple-input multiple-output
MNO	Mobile network operator
MXR	Medical extended reality
NR	New Radio
NSA	Non-standalone
NSF	National Science Foundation
NUT	Network under test
OSEL	Office of Science and Engineering Laboratories
OTA	Over-the-air
PAA	Phased antenna array
PAWR	Platforms for Advanced Wireless Research
PHY	Physical (layer)
QoS	Quality of Service
RAN	Radio access network
RF	Radio Frequency
RPC	Remote procedure call
RRH	Remote radio head
RRM	Radio Resource Management
RST	Regulatory science tool
RTT	Round-trip time
S1AP	S1 (interface) application protocol (3GPP)
SA	Standalone
SDO	Standard development organization
SDR	Software-defined radio
SW	Software
TAP	Test access point
TCP	Transmission control protocol
TRUST	Testbed as a regulatory science tool
TRX	Transceiver
UDP	User datagram protocol
UE	User equipment
UE-GW	UE as a Gateway (to 5G RAN)
UI	User Interface
UL	Uplink (from UE to RAN)
VR	Virtual reality
WebEM	Web element manager
XR	Extended reality

**TABLE 2:** Examples of 5G system components and their features in testbed implementations

	Real Case (R)	Emulation (E)	Simulation (S)
Overall	+ Ground truth + Rich in details & side info - High implementation cost - Limited visibility and controllability - Costly RF environment controls - Slow to incorporate novel features	+ Mixed HW/SW advances + Faster than computer simulation - Generic HW often slower than dedicated HW - Need training for running user-defined specifications	+ Fast deployment + Simplified system behavior for high impact factors + Test at scale + Repeatability + Cost efficient - Modeling accuracy - Often slower than real/emulated cases
UE	*Smartphones, FWA, chipset dev. kits* + Real OTA 5G connectivity - Slow to incorporate novel features	*SDRs, (multi-)UE emulators* + OTA tests - Single unit emulators do not capture spatial distribution	*UE protocol stack simulators* + Standard traffic generation - Limited testing with over-the-top apps
5G Channel	*OTA test fields, trial sites, shielded enclosures* + Accurate channel responses - Cost to access test locations - Might require spectrum licensing	*Channel emulators* + Tunable channel coeff. - Support a limited number of channels/users	*Simulation channel models* + Flexible channel options - Partial adherence to real channel responses
RAN	*Carrier-grade eNB/gNB* + Realistic insight into real network operations - Vendor-specific proprietary systems, support & configurations	*SDRs, RAN emulators* + Connect to real UE w/fast deployment - RF limitations per HW/SW specifications	*RAN simulators* + Cost-effective for multi-cell tests - Only implement selected User/Control Plane behaviors & abstraction
Core	*Carrier-grade Core* + Realistic insight into real network operations - Costly - Might require additional operation tools	*Core emulators* + Lite installation & maintenance cost - Limited functions per HW/SW specs	*Core function libraries from* *network simulator suites* + Support mobility tests w/basic RAN management - Oversimplified RAN-Core interface w/non-standard messages
DN	*ISP + Cloud providers* + Real Internet traffic fluctuations + Testing Cloud-native applications w/original service engines - Internet access constraints per IT security policies	*Intranet server clusters* + Air-gapped tests w/full information control - Testing Cloud applications w/o platform accelerators - Steep learning curve for Cloud app developers	*Internet simulators, e.g., BGP routing* + Large network-scale tests in “sand box” - Limited implementation

The + sign refers to the advantages and the - sign refers to the disadvantages of a given implementation option.

**TABLE 3:** Example profiles for assembling 5G NUT in test tasks^a^.

Test Scale		MD User	5G Connectivity					MD Remote	Example
			UE	Channel	RAN	Core	DN		
Cat. 1	MD Function Test	Function < − − − − − − − − − − − − −− > Function							
1.1	5G compatible MD	R	R/E/S					R	[11]
1.2	5G built-in MD	R		R/E/S				R	[11]
1.3	5G MD field trial	R	R					R	[5], [6]
Cat. 2	5G Ntwk E2E Test		Node < − − − − − − − − − − − > Node						
2.1	UE acceptance test/Network survey		R	R	R	R	R		[9], [34]
2.2	UE conform. test		R	E	E	E			[9], [35]
2.3	NR protocol verification		E	R (OTA)	E	E			[36]
2.4	UE prototyping		E	\	S	S			[37]
2.5	Core function test		S	S	S	E	R/E		[38]
2.6	5G RRM verification		S	S	S	S	S		[39]
Cat. 3	5G RF Link Test		TRX < − − − − −− > TRX						
3.1	Emission test	R/E	R	R (OTA)	E				[40], [41]
3.2	5G PHY verification		E	R (OTA)	E				[42], [43]
3.3	5G link analysis		S	S	S				[44], [45]

a Real is abbreviated by R, emulated by E, simulated by R, medical device by MD, user equipment by UE, data network by DN, end-to-end by E2E, radio resource management by RRM, physical layer by PHY, and over-the-air by OTA, transceiver by TRX.

**TABLE 4:** FDA/CDRH/OSEL 5G testbed technical specifications

	Equipment	Hardware	5G/RF Specs	Software
UE	Samsung S21 Ultra (R)	Qualcomm Snapdragon X60 chipset	NSA: 2, 5, 12, 25, 30, 41, 48, 66, 71, 77, 78, 260, 261; SA: 2, 5, 12, 30, 41, 48, 66, 71, 77, 78	Android 12
5G Channel	OTA Indoor (R)	Anechoic chamber	Dim. 40′(L)x25′(W)x27′(H) RF shielded (1–60 GHz)	N/A
RAN	Nokia AirScale eNB (R)	ASIA LTE Common ABIA LTE Capacity	LTE B66 (20 MHz FDD, AHFIB Sub-6 GHz Dual RRH, 4T4R)	5G21B/2137
	Nokia AirScale gNB (R)	ASIB 5G Common ABIL 5G Capacity (FR1) ABIL 5G Capacity (FR2)	NR n2 (20 MHz FDD, AHFIB Sub-6 GHz Dual RRH, 4T4R), NR n260 (100 MHz TDD, AEWD mmWave RRH, 2T2R, 64 AE, 16 Beams/AP x 2 APs)	5G21B/2137
Core	Valid8 mobile core network emulator M5 (E)	NSA/SA core emulator (Firmware Ver 8.6.74)	5G NSA/SA — 3GPP R15	Valid8 Proprietary
DN	IMS server (E)	Intel NUC8BEK	Kamailio SIP server Ver 5.1.2	Ubuntu 18.04 LTS
	Application server (R)	Dell Precision 7760	MXR server, HTTP server	Win 11 Pro

**TABLE 5:** Select panel functions and configurations in the 5G testbed for MXR demo

	Application	Functions	Equipment	Software Tools
DevNet Panel	MXR Server (R)	Virtual host & client hub	Dell Precision 7760 (Win 11 Pro)	Unreal Engine Ver 4.27.2
		Local medical image server		Apache server Ver 2.4
	MXR Client 1 (R)	Virtual attendant	Microsoft Hololens2	Unreal Engine Ver 4.27.2
	MXR Client 2 (R)	Virtual attendant	Dell Latitude E series (Win 10 Pro)	Unreal Engine Ver 4.27.2
	5G connectivity (R/E)	Client-server communications	See Table 4	See Table 4
CTRL Panel	UE remote access & control	Radio ON/OFF, status Monitor., link quality test, UE LAN mgmt	Dell Latitude E series (Ubuntu 18.04 LTS)	Android 12 + ADB + scrcpy v1.2.5
	RAN remote access & control	eNB/gNB ON/OFF, BTS & cell mgmt, SW version control	Dell Precision M6800	Nokia Web Element Manager (WebEM) Ver 57354
	Core remote access & control	ON/OFF control, environ. config.		Valid8 WebUI for ProtocolEngine Ver 8.6.74
MEAS Panel	5G link at UE (as a gateway)	IP flow metrics, e.g., throughput, latency.	Dell Latitude E series (Ubuntu 18.04 LTS)^a^	Termux Ver 0.118.0 + iPerf3
		UE IP traffic captur.	SharkTAP CC + Dell Precision 7750 (Ubuntu 18.04 LTS)	Wireshark
	Server end performance	Server IP traffic captur.		
	RAN status & traffic	BTS performance counters, IP traffic captur.	Dell Precision M6800^b^	Nokia WebEM Ver 57354
	Core status & traffic	M5 snapshot, core network element IP traffic captur.		Valid8 WebUI & Tcpdump @M5 over SSH

a,b The equipment can serve multiple functions across panels if operating within capacity.

**TABLE 6:** Latency and throughput performance in connected 5G links

UE	Latency (RTT in msec) min/avg/max/std	Throughput (Mbps)		
			TCP	UDP
Pos. #1 (GW of HL2)	13.003/24.368/120.765/10.455	DL	76.1	217
		UL	50.6	58.5
Pos. #2 (GW of WinC)	14.507/20.432/45.117/3.852	DL	84.8	>400
		UL	60.0	129
